# Associations between future orientation and motivated learning: the roles of AI self-efficacy and intrinsic motivation among Chinese university students

**DOI:** 10.3389/fpsyt.2026.1842858

**Published:** 2026-07-15

**Authors:** Ying Luo, Jianwen Chen, Hechen Li, Ronghua Zhang

**Affiliations:** 1 Graduate School of Education, Huazhong University of Science and Technology, Wuhan, China; 2 Department of Ideological and Political Education, School of Marxism, Wuhan University, Wuhan, China

**Keywords:** AI self-efficacy, future orientation, intrinsic motivation, motivated learning, undergraduates

## Abstract

**Introduction:**

As AI-related technologies and products become increasingly accessible in higher education, it is important to understand how students' AI-related competence beliefs are associated with motivated learning. Guided by the motivation-cognition-behavior perspective and self-determination theory, this study examined whether future orientation was associated with university students' motivated learning through AI self-efficacy and intrinsic motivation.

**Methods:**

A cross-sectional, face-to-face survey was conducted with 431 university students at a university in Hubei Province, China, using classroom-based group recruitment. Hayes' PROCESS macro Model 6 with 5,000 bias-corrected bootstrap samples was used to test the theoretically specified indirect pathways.

**Results:**

The results showed that future orientation was positively associated with motivated learning, with a standardized total effect of β = .549. The total indirect effect through AI self-efficacy and intrinsic motivation was also significant, effect = .196, 95% CI [.129, .274]. Bootstrap analyses further indicated that AI self-efficacy and intrinsic motivation statistically accounted for indirect associations between future orientation and motivated learning, both separately and in a theoretically specified serial pathway. The indirect association through intrinsic motivation was numerically larger than that through AI self-efficacy, although this difference should be interpreted cautiously given the cross-sectional design.

**Discussion:**

The findings provide preliminary evidence that future orientation, AI-related competence beliefs, and intrinsic motivation are relevant to students' motivated learning toward professional skill acquisition in university contexts where AI-related tools are increasingly accessible. Theoretical and practical implications are discussed.

## 1 Introduction

### 1.1 Background

Motivated learning toward professional skill acquisition is an important condition of successful university study because it reflects students’ willingness, effort, determination, and perceived importance in acquiring professional skills (1). In contemporary higher education, students are increasingly expected to update professional knowledge, develop transferable competencies, and adapt to changing academic and career demands. These expectations have become more salient in university contexts where generative AI tools and related digital resources are increasingly accessible to students. However, the growing availability of AI-related technologies does not automatically lead to stronger learning engagement. Rather, it raises an important motivational question: which learner characteristics and psychological processes are associated with students’ motivated learning toward professional skill development?

Future orientation is one learner characteristic that may be particularly relevant to motivated learning. It refers to a relatively stable tendency to anticipate, evaluate, and prepare for desired future outcomes (2, 3). Students with stronger future orientation may be more likely to connect present academic effort with long-term academic or career goals, regulate their behavior in ways that support these goals, and persist when learning requires sustained effort or delayed gratification (4, 5). Prior research has linked future orientation to adaptive academic outcomes, including engagement, self-regulation, persistence, and academic adjustment (6–9). Nevertheless, less is known about how future orientation is associated with motivated learning specifically directed toward professional skill acquisition, especially in university contexts where AI-related tools are becoming increasingly visible.

AI self-efficacy may represent one important competence-related process in this context. Self-efficacy theory suggests that learners’ perceived capability shapes how they interpret challenges, regulate effort, and persist in demanding situations (10). Extending this logic to AI-accessible learning environments, AI self-efficacy refers to students’ perceived confidence and ease in learning about, interacting with, and making AI technologies/products perform intended tasks (11). This construct differs from general digital self-efficacy because AI-related technologies often involve adaptive responses, conversational interaction, perceived uncertainty, and task-control demands. Therefore, students’ perceived competence in relation to AI technologies/products may be psychologically relevant to motivated learning, not because AI self-efficacy is the primary outcome of interest, but because it may help explain how students respond to contemporary learning demands involving emerging technologies.

Intrinsic motivation is another key motivational process that may help explain motivated learning. According to self-determination theory, intrinsic motivation refers to engaging in learning because of interest, curiosity, enjoyment, and inherent satisfaction (12). Although intrinsic motivation and motivated learning are related, they are not equivalent. Intrinsic motivation concerns why students engage in learning, whereas motivated learning toward professional skill acquisition concerns the extent to which students are willing to invest effort, show determination, and attach importance to acquiring professional skills. Students who find learning personally meaningful and interesting may be more likely to sustain effort and persist in professional skill development. Thus, intrinsic motivation may serve as a proximal motivational process linking future-oriented goals and competence-related beliefs to motivated learning.

Taken together, the literature suggests three unresolved issues. First, although future orientation has been linked to adaptive academic outcomes, its association with professional skill-oriented motivated learning remains insufficiently specified. Second, although AI self-efficacy has received increasing attention, its role within broader motivational processes has not been fully clarified. Third, although intrinsic motivation is central to learning engagement, limited research has integrated future orientation, AI-related competence beliefs, intrinsic motivation, and motivated learning within one framework. Addressing these gaps, the present study examines whether future orientation is associated with motivated learning through AI self-efficacy and intrinsic motivation among Chinese university students.

### 1.2 Theoretical framework and hypothesis development

#### 1.2.1 Theoretical foundation

The present study is guided by a motivation-cognition-behavior logic and self-determination theory. The motivation-cognition-behavior logic provides a useful organizing framework for understanding how relatively distal motivational orientations may be associated with learning-related outcomes through cognition-related beliefs and proximal motivational processes (10, 12). Within this framework, future orientation is positioned as a distal motivational orientation because it reflects how students connect present learning with anticipated future goals (2, 3). AI self-efficacy is positioned as a cognition-related competence belief because it captures students’ perceived confidence and capability in engaging with AI technologies/products in learning contexts (11). Intrinsic motivation is positioned as a proximal motivational process because it reflects interest, enjoyment, curiosity, and self-endorsed engagement in learning (12). Motivated learning toward professional skill acquisition is positioned as the outcome variable because it reflects students’ willingness, effort, determination, and perceived importance in acquiring professional skills (1).

Self-determination theory further helps explain why competence-related beliefs and intrinsic motivation may be relevant to motivated learning. From this perspective, students are more likely to engage actively in learning when they experience a sense of competence and when learning is experienced as interesting, meaningful, or self-endorsed (12–14). Therefore, AI self-efficacy is not treated as the primary focus of the study, but as one domain-specific competence belief that may help explain how students respond to AI-accessible learning environments. Intrinsic motivation, in turn, represents a more proximal motivational process through which future-oriented goals and competence beliefs may be associated with students’ motivated learning. On this basis, the present study examines both the direct association between future orientation and motivated learning and the theoretically specified indirect associations through AI self-efficacy and intrinsic motivation.

#### 1.2.2 Future orientation and motivated learning

Future orientation may be positively associated with motivated learning toward professional skill acquisition. Future orientation reflects students’ tendency to anticipate, evaluate, and prepare for desired future outcomes (2, 3). In university learning contexts, this future-directed orientation may help students interpret present academic tasks as meaningful steps toward long-term academic, professional, and personal development. When students perceive current learning as relevant to their future goals, they are more likely to attach importance to professional skill acquisition and to invest sustained effort in learning activities.

This relationship is especially relevant because motivated learning toward professional skill acquisition requires more than temporary interest. It involves willingness, effort, determination, and the perceived importance of acquiring professional skills (1). Students with stronger future orientation may be better able to regulate present effort in light of delayed benefits, persist when learning is difficult, and view professional skill development as personally valuable. Prior research has similarly shown that future-oriented students tend to demonstrate stronger academic engagement, self-regulation, persistence, and academic adjustment (4–7). Therefore, future orientation is expected to be positively associated with motivated learning.

H1: Future orientation is positively associated with motivated learning.

#### 1.2.3 AI self-efficacy as a competence-belief pathway

AI self-efficacy may serve as a competence-belief pathway linking future orientation to motivated learning. According to self-efficacy theory, learners’ perceived capability shapes how they interpret challenges, regulate effort, and persist in demanding situations (10). In the present study, AI self-efficacy refers to students’ perceived confidence and ease in learning about, interacting with, and making AI technologies/products perform intended tasks (11, 15). This construct is not treated as a direct indicator of actual AI exposure, intensity of AI use, or formal AI integration in coursework. Rather, it reflects students’ perceived capability to deal with AI-related technologies/products in learning contexts.

Future orientation may be positively associated with AI self-efficacy. Students with stronger future orientation are more likely to view present learning demands in relation to long-term academic and career development. In contemporary university contexts, AI-related technologies/products may be perceived as part of the changing skill environment that students need to understand and manage. Future-oriented students may therefore be more willing to explore unfamiliar learning tools, invest effort in understanding AI-related technologies, and interpret AI-related competence as relevant to their future development. Such goal-directed engagement may be associated with stronger perceived capability in learning about and interacting with AI technologies/products.

AI self-efficacy may also be positively associated with motivated learning. When students feel more capable of engaging with AI-related technologies/products, they may experience less uncertainty and greater perceived control in learning contexts where such tools are increasingly visible. This perceived capability may make demanding learning tasks appear more manageable, support persistence, and strengthen students’ willingness to invest effort in professional skill acquisition. In this sense, AI self-efficacy may function as a domain-specific competence belief that helps explain why some students are more willing to sustain motivated learning in AI-accessible university environments.

Taken together, future-oriented students may report stronger AI self-efficacy, and this competence belief may in turn be associated with stronger motivated learning toward professional skill acquisition. Accordingly, the following hypothesis was proposed:

H2: Future orientation is indirectly associated with motivated learning through AI self-efficacy.

#### 1.2.4 Intrinsic motivation as an internal motivational pathway

Intrinsic motivation may represent another theoretically meaningful pathway linking future orientation to motivated learning. According to self-determination theory, intrinsic motivation refers to engaging in learning because of interest, enjoyment, curiosity, and inherent satisfaction (12). In the present study, intrinsic motivation is treated as an internal motivational source, whereas motivated learning is treated as a learning-related tendency involving willingness, effort, determination, and the perceived importance of acquiring professional skills (1). Thus, although the two constructs are conceptually related, they are not equivalent. Intrinsic motivation emphasizes why students engage in learning, whereas motivated learning emphasizes the extent to which students are willing to invest effort in professional skill acquisition.

Future orientation may be positively associated with intrinsic motivation. Students with stronger future orientation may be more likely to perceive current academic work and professional skill development as connected to valued long-term goals. When present learning is understood as personally meaningful and relevant to future development, students may be more likely to experience learning as self-endorsed rather than externally imposed. This perceived meaning and goal congruence may support greater interest, curiosity, enjoyment, and autonomous engagement in learning (4, 12).

Intrinsic motivation may also be positively associated with motivated learning. Students who learn out of interest and inherent satisfaction are more likely to sustain effort, show determination, and attach greater importance to acquiring professional skills. Prior research has shown that intrinsic motivation is associated with deeper engagement, persistence, and more active participation in learning contexts (13, 14). Therefore, intrinsic motivation may serve as a proximal internal motivational process through which future orientation is associated with motivated learning toward professional skill acquisition.

Accordingly, the following hypothesis was proposed:

H3: Future orientation is indirectly associated with motivated learning through intrinsic motivation.

#### 1.2.5 The serial pathway from AI self-efficacy to intrinsic motivation

AI self-efficacy and intrinsic motivation may also operate as a theoretically specified serial pathway linking future orientation to motivated learning. As discussed above, future-oriented students may be more likely to develop stronger perceived competence in relation to AI technologies/products, and such AI self-efficacy may support motivated learning by reducing uncertainty and strengthening perceived control. However, competence beliefs may also be linked to motivated learning through a more proximal motivational process.

From the perspective of self-determination theory, perceived competence is an important condition for more autonomous and interest-based engagement in learning (12). When students feel capable of understanding and interacting with AI technologies/products, they may be more likely to approach AI-accessible learning environments with curiosity, exploration, and initiative rather than avoidance or uncertainty. In this sense, AI self-efficacy may support intrinsic motivation by making learning experiences feel more manageable, meaningful, and self-endorsed. This intrinsic motivation may then be associated with stronger motivated learning because students who experience greater interest and enjoyment are more likely to sustain effort and attach importance to professional skill acquisition.

Accordingly, the present study positions AI self-efficacy upstream of intrinsic motivation in a theoretically specified sequence. Future orientation may be associated with stronger AI self-efficacy, which may in turn be associated with greater intrinsic motivation and, subsequently, stronger motivated learning. Given the cross-sectional design, this ordering is interpreted as a theoretically grounded indirect association rather than as evidence of a confirmed temporal process. With this caution in mind, the following hypothesis was proposed:

H4: Future orientation is indirectly associated with motivated learning through the theoretically specified serial pathway of AI self-efficacy and intrinsic motivation.

### 1.3 The present study

**Figure 1:**
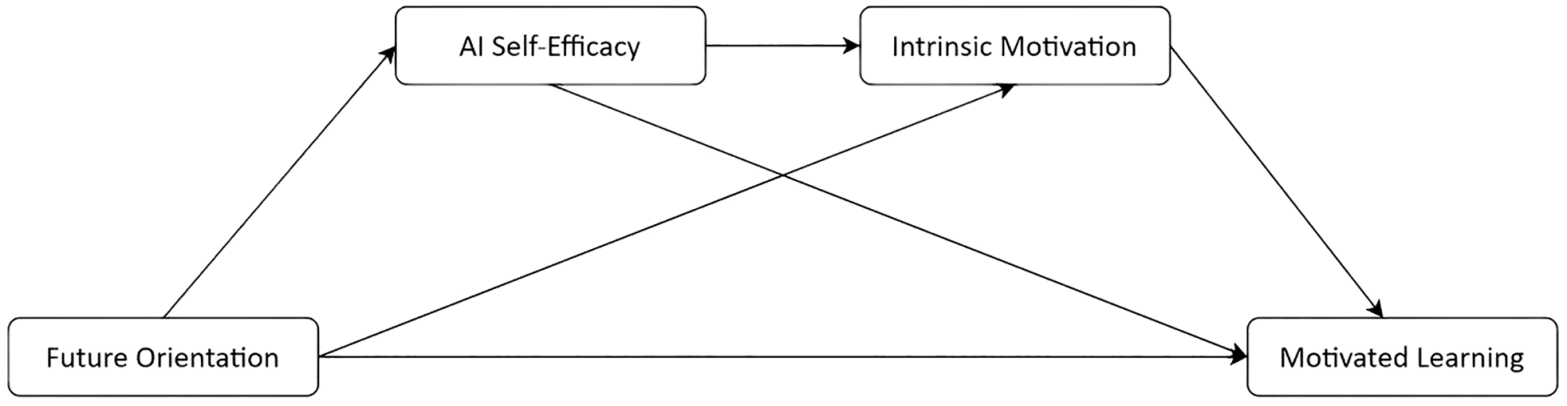
Research framework.

The present study integrates future orientation, AI self-efficacy, intrinsic motivation, and motivated learning within a single explanatory framework relevant to university learning. Specifically, future orientation is conceptualized as a distal motivational orientation, AI self-efficacy as a cognition-related competence belief, intrinsic motivation as a proximal motivational process, and motivated learning toward professional skill acquisition as the focal outcome (**Figure 1**). This framing allows the study to examine AI self-efficacy as part of a broader motivational process rather than as an isolated AI-related construct.

Accordingly, this study examined whether future orientation was associated with motivated learning among Chinese university students and whether this association was statistically accounted for by AI self-efficacy and intrinsic motivation. The study tested one direct association and three theoretically specified indirect associations: the direct association between future orientation and motivated learning, the indirect association through AI self-efficacy, the indirect association through intrinsic motivation, and the serial indirect association through AI self-efficacy and intrinsic motivation. Given the cross-sectional design, these pathways were interpreted as theoretically specified indirect associations rather than evidence of temporal or causal mediation.

## 2 Methods

### 2.1 Participants

Participants were recruited from a university in Hubei Province, China through classroom-based group recruitment during scheduled in-person lecture sessions. Data were collected during a period when generative AI tools were widely accessible to university students. The study focused on students’ perceived AI-related competence rather than on direct behavioral tracking of actual AI use. The questionnaires were administered face to face during the lecture sessions, and all participants completed the survey under the supervision of trained researchers.

A total of 437 questionnaires were collected. To improve transparency, we report the number of cases excluded at each screening step. Responses that failed either of the two attention-check items were excluded during data screening. After screening, 431 valid questionnaires were retained, yielding a valid response rate of 98.6%.

The final sample consisted of 431 university students, including 170 male students and 261 female students. Participants ranged in age from 19 to 30 years, with a mean age of 23.15 years (*SD* = 1.53). In terms of educational level, 388 participants were undergraduate students (90.0%) and 43 were graduate students (10.0%). Regarding major category, the sample included students from humanities and social sciences (n = 179, 41.5%), science-related disciplines (n = 39, 9.0%), engineering (n = 138, 32.0%), medicine (n = 39, 9.0%), agriculture (n = 21, 4.9%), and arts-related disciplines (n = 15, 3.5%).

### 2.2 Measures

#### 2.2.1 Future orientation (FO)

FO was assessed using the Future-Oriented Coping Scale developed by Gan (16). The scale contains 16 items across two dimensions, preventive coping and proactive coping, and was used in the present study to capture a relatively stable future-directed orientation toward anticipated demands and goals. A sample item is, “Before taking action, I develop strategies to improve situations I want to change.” Items were rated on a 4-point Likert scale ranging from 1 (strongly disagree) to 4 (strongly agree), with higher scores indicating stronger future orientation. In the present study, the scale showed acceptable internal consistency (Cronbach’s α = .78).

#### 2.2.2 AI self-efficacy (AISE)

AISE was measured using five items adapted from Wang and Chuang (11). In the present study, AI self-efficacy refers to students’ perceived confidence, perceived ease, and efficacy-related beliefs in learning about and interacting with AI technologies/products. A sample item is, “I find it easy to make AI technologies/products perform the tasks I want them to complete”. Responses were recorded on a 7-point Likert scale ranging from 1 (strongly disagree) to 7 (strongly agree), with higher scores indicating greater AI self-efficacy. The scale demonstrated acceptable reliability in this study (Cronbach’s α = .75).

#### 2.2.3 Intrinsic motivation (IM)

IM was assessed using the relevant subscale of the Learning Motivation Scale revised by Chi (17), originally developed by Amabile (18). The subscale includes 14 items and was used to capture learning driven by interest, curiosity, enjoyment, and inherent satisfaction. A sample item is, “I do many things driven by curiosity.” Participants responded on a 4-point Likert scale ranging from 1 (disagree) to 4 (agree), with higher scores indicating stronger intrinsic motivation. In the present study, the scale demonstrated good internal consistency (Cronbach’s α = .81).

#### 2.2.4 Motivated learning (ML)

ML was measured using the five-item scale developed by Wang and Wang (1). The scale captures students’ willingness, effort, determination, and perceived importance in professional skill-oriented motivated learning. A representative item is, “I am willing to work hard to learn another professional skill”. Items were rated on a 7-point Likert scale ranging from 1 (strongly disagree) to 7 (strongly agree), with higher scores indicating stronger motivated learning. The scale demonstrated good reliability in this study (Cronbach’s α = .89).

#### 2.2.5 Demographic variables

Demographic information collected included sex, age, major, and education level.

### 2.3 Data analysis

Data were analyzed using SPSS 27.0 and JASP. Descriptive statistics, Pearson correlations, and reliability analyses were first conducted. A parcel-level confirmatory factor analysis was then performed in JASP to examine the measurement structure and discriminant validity of the four focal constructs. Parcel-level indicators were used rather than item-level indicators because several constructs contained multiple items, and the CFA was intended to provide preliminary evidence for the distinctiveness of the four focal constructs before the observed-variable PROCESS analysis. This approach helped reduce model complexity and aligned the measurement analysis with the subsequent indirect-effect analysis based on composite scores. Parcels were created based on the theoretical structure of the scales and balanced item allocation, rather than on *post hoc* modification indices. Model fit was evaluated using χ^2^/df, CFI, TLI, RMSEA, and SRMR. Composite reliability, average variance extracted, the Fornell–Larcker criterion, and HTMT ratios were also examined.

Common method bias was assessed using Harman’s single-factor test as a preliminary diagnostic. Procedural remedies, including anonymous responses, standardized instructions, voluntary participation, and attention-check items, were also adopted during data collection to reduce potential common method bias. Finally, Hayes’ PROCESS macro Model 6 was used to test the theoretically specified serial indirect effects, with gender, age, major, and education level included as covariates to adjust for potential demographic differences in university learning experiences. These covariates were not part of the theoretical framework and were therefore not interpreted as focal predictors. All focal variables were standardized, and indirect effects were tested using 5,000 bootstrap samples with 95% bias-corrected confidence intervals. Because PROCESS uses observed composite scores, the results are interpreted as observed-variable indirect associations rather than latent-variable causal effects.

## 3 Results

### 3.1 Measurement model and discriminant validity

Before testing the indirect effects, a parcel-level confirmatory factor analysis was conducted to examine whether the four focal constructs could be empirically distinguished. As described in the Data Analysis section, a parcel-level CFA was conducted to provide preliminary evidence for the empirical distinctiveness of the four focal constructs. The four-factor parcel-level model showed acceptable fit to the data, χ^2^(59) = 210.80, *p* <.001, χ^2^/df = 3.57, CFI = .944, TLI = .926, RMSEA = .077, 90% CI [.066,.089], and SRMR = .054. All standardized parcel loadings were statistically significant and ranged from.558 to.905.

Composite reliability values ranged from.763 to.906, indicating acceptable internal consistency at the construct level. AVE values ranged from.454 to.762, with most values above or close to the conventional.50 threshold. Discriminant validity was further examined using the Fornell–Larcker criterion and HTMT ratios. The HTMT ratios ranged from.481 to.663, all below the conservative threshold of.85. Importantly, the HTMT value between IM and ML was.574, suggesting that these two motivational constructs were related but empirically distinguishable. Overall, the measurement results provided preliminary support for the reliability and empirical distinctiveness of the four constructs.

### 3.2 Common method bias test

Given the use of self-report measures, common method bias was examined using Harman’s single-factor test as a preliminary diagnostic (19). An unrotated exploratory factor analysis extracted 10 factors with eigenvalues greater than 1. The first factor accounted for 24.65% of the total variance, which was below the commonly used threshold of 40%. This result suggests that no single factor dominated the covariance structure of the measures. In addition, several procedural remedies were adopted to reduce potential common method bias, including anonymous responses, standardized instructions, voluntary participation, and attention-check items.

### 3.3 Descriptive statistics and correlation analysis

Descriptive statistics and bivariate correlations among the key variables are presented in **Table 1**. Overall, participants reported relatively high levels of FO, AISE, IM, and ML, as the mean scores of all four variables were above the midpoint of their respective response scales. The correlation results showed that FO was positively associated with AISE (*r* = .36, *p* <.01), IM (*r* = .54, *p* <.01), and ML (*r* = .55, *p* <.01), suggesting that students with stronger future orientation tended to report higher AI-related efficacy beliefs, stronger intrinsic motivation, and greater motivated learning. AISE was also positively associated with IM (*r* = .43, *p* <.01) and ML (*r* = .44, *p* <.01), indicating that students who felt more confident in learning about and interacting with AI technologies/products also tended to report stronger motivational outcomes. In addition, IM was positively correlated with ML (*r* = .51, *p* <.01). This moderate correlation suggests that the two constructs were related but not redundant, which is consistent with the discriminant validity evidence reported above. Taken together, these correlation patterns were consistent with the theoretically specified indirect-effect model and provided a preliminary basis for further analysis.

**Table 1:** Means, standard deviations, and correlations among key variables.

Variables	Descriptive statistics		Correlations			
	Mean	SD	1	2	3	4
1.FO	2.93	0.31	1			
2.AISE	5.23	0.86	0.36^**^	1		
3.IM	3.07	0.40	0.54^**^	0.43^**^	1	
4.ML	5.12	1.03	0.55^**^	0.44^**^	0.51^**^	1

AISE, AI self-efficacy; IM, intrinsic motivation; ML, motivated learning. ^**^p <.01; the same notations apply below.

### 3.4 Serial indirect effect analysis

A serial indirect-effect analysis was conducted using Hayes’ PROCESS macro Model 6. Consistent with the hypotheses, the analysis focused on the paths among future orientation, AI self-efficacy, intrinsic motivation, and motivated learning. Gender, age, major, and education level were included as covariates.

**Figure 2:**
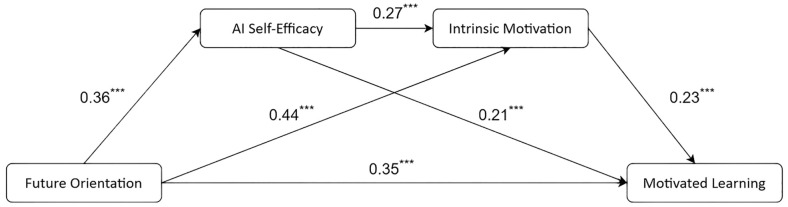
Standardized path coefficients in the serial indirect-effect model. ***p < .001.

As shown in **Table 2** and illustrated in **Figure 2**, FO was positively associated with AISE, *β* = .355, *p* <.001. FO and AISE were both positively associated with IM, *β* = .438, *p* <.001, and *β* = .266, *p* <.001, respectively. In the final equation predicting ML, FO, AISE, and IM were all significant positive predictors, *β* = .353, *p* <.001; *β* = .211, *p* <.001; and *β* = .227, *p* <.001, respectively. These results suggest that students with stronger future orientation tended to report higher AI self-efficacy, stronger intrinsic motivation, and higher motivated learning.

**Table 2:** Regression results for the serial indirect effect model.

Outcome	Predictor	*β*	*t*	*p*	Outcome
AISE	FO	.355	7.835	<.001	AISE
IM	FO	.438	10.503	<.001	IM
IM	AISE	.266	6.375	<.001	IM
ML	FO	.353	7.886	<.001	ML
ML	AISE	.211	5.053	<.001	ML
ML	IM	.227	4.890	<.001	ML

The bootstrap results are presented in **Table 3**. The total indirect effect of FO on ML through AISE and IM was significant, effect = .196, Boot SE = .037, 95% CI [.129,.274]. Three specific indirect pathways were identified. First, FO was indirectly associated with ML through AISE, effect = .075, Boot SE = .021, 95% CI [.037,.120]. Second, FO was indirectly associated with ML through IM, effect = .100, Boot SE = .024, 95% CI [.055,.152]. Third, the theoretically specified serial indirect pathway through AISE and IM was also significant, effect = .021, Boot SE = .009, 95% CI [.008,.043]. The total effect of FO on ML was *β* = .549, and the direct effect remained significant after including AISE and IM, *β* = .353, indicating a partial indirect association.

**Table 3:** Bootstrap results for indirect effects.

Path	Standardized indirect effect	Boot SE	95% CI	Proportion of total effect
Total indirect effect	.196	.037	[.129,.274]	35.70%
FO → AISE → ML	.075	.021	[.037,.120]	13.66%
FO → IM → ML	.100	.024	[.055,.152]	18.03%
FO → AISE → IM → ML	.021	.009	[.008,.043]	3.83%

Bootstrap confidence intervals were based on 5,000 bootstrap samples. Confidence intervals that do not include zero indicate statistically significant indirect effects.

The relative proportions of the indirect effects are reported in **Table 3** as descriptive indicators only. Given the cross-sectional design, these proportions should be interpreted cautiously and were not used as the primary basis for evaluating the indirect pathways. Full regression results including demographic covariates are provided in **Supplementary Table 1**.

## 4 Discussion

The psychological processes linking future orientation to motivated learning remain insufficiently specified in university contexts where AI-related technologies/products are becoming increasingly accessible. Using cross-sectional survey data from 431 Chinese university students, this study examined whether future orientation was associated with motivated learning toward professional skill acquisition and whether this association was statistically accounted for by AI self-efficacy and intrinsic motivation. Overall, the findings were consistent with the proposed motivation-cognition-behavior logic and self-determination theory. Future orientation was positively associated with motivated learning, suggesting that students’ future-directed goals may help them interpret present professional skill learning as meaningful and worth sustained effort. AI self-efficacy and intrinsic motivation further accounted for significant indirect associations, indicating that future orientation may be linked to motivated learning not only through students’ general future-directed goal orientation, but also through their perceived competence in AI-accessible learning environments and their self-endorsed interest in learning. In this sense, the findings suggest a layered motivational process: distal future-oriented goals are associated with cognition-related competence beliefs and proximal intrinsic motivation, which together relate to students’ motivated learning. Given the cross-sectional design, these findings should be interpreted as theoretically grounded associations rather than evidence of temporal or causal mediation.

The positive association between future orientation and motivated learning suggests that students’ future-directed thinking may provide an important motivational basis for professional skill acquisition. This finding is consistent with prior research linking future orientation to adaptive academic outcomes, including engagement, self-regulation, persistence, and academic adjustment (4–9). More importantly, the finding helps explain why future orientation may matter for motivated learning. Students with stronger future orientation may be more likely to interpret present academic tasks as meaningful investments in their future academic and professional development. As a result, professional skill learning may be experienced not merely as a current academic requirement, but as a purposeful step toward future goals.

This interpretation is consistent with the motivation-cognition-behavior logic adopted in the present study. Future orientation, as a distal motivational orientation, may shape how students assign meaning to present learning activities and regulate their current effort in light of anticipated future outcomes (2, 3). When students perceive professional skill acquisition as relevant to their future self-development, they may be more willing to persist in learning even when the benefits are delayed or when the learning process is demanding. Therefore, the present finding extends prior work by showing that future orientation is not only related to general academic adjustment or engagement, but also to motivated learning toward professional skill acquisition in AI-accessible university contexts (1, 20, 21). This supports the view that future orientation is a meaningful learner characteristic for understanding why some students maintain stronger willingness, effort, and determination in professional skill learning.

AI self-efficacy also statistically accounted for part of the association between future orientation and motivated learning. This pathway can be understood through self-efficacy theory, which emphasizes that perceived capability shapes how learners interpret challenges, regulate effort, and persist in demanding situations (10). Students with stronger future orientation may be more attentive to the skills and competencies that are likely to matter for their future academic or professional development. In university contexts where AI-related technologies/products are increasingly accessible, such students may be more likely to regard AI-related competence as relevant to their future learning and career preparation. This future-oriented interpretation may help explain why stronger future orientation was associated with higher AI self-efficacy.

The link between AI self-efficacy and motivated learning may reflect the role of perceived control in contemporary learning environments. Students who feel more capable of learning about and interacting with AI technologies/products may experience AI-accessible learning contexts as more manageable and less uncertain. This sense of competence may reduce hesitation when facing unfamiliar tools or technology-supported learning demands and may help students sustain effort in professional skill acquisition. Therefore, AI self-efficacy appears to function as a cognition-related competence belief within the motivation-cognition-behavior framework: future-oriented goals may be associated with stronger perceived AI-related capability, which in turn is related to greater willingness and effort in motivated learning.

At the same time, this interpretation should remain cautious. The present study measured perceived AI-related competence rather than actual AI use, AI exposure, or formal AI integration in coursework. Thus, the findings do not indicate that AI use itself improves motivated learning. Instead, they suggest that students’ beliefs about their capability to deal with AI-related technologies/products may be psychologically relevant to professional skill-oriented motivated learning in AI-accessible university contexts (11, 15, 20).

Intrinsic motivation also statistically accounted for part of the association between future orientation and motivated learning, which provides further support for the role of proximal motivational processes in the proposed framework. From the perspective of self-determination theory, future orientation may contribute to motivated learning not only by directing students toward long-term goals, but also by helping them experience present learning as personally meaningful and self-endorsed (12). Students with stronger future orientation may be more likely to understand academic work and professional skill development as connected to valued future possibilities. When this connection is internalized, learning may be experienced less as an external requirement and more as an activity that is meaningful, interesting, and aligned with students’ own goals.

This interpretation helps explain why intrinsic motivation was associated with motivated learning. Unlike AI self-efficacy, which reflects students’ perceived capability in relation to AI technologies/products, intrinsic motivation reflects interest, enjoyment, curiosity, and self-endorsed engagement in learning. Such internal motivation may provide the psychological energy needed to sustain effort, show determination, and attach importance to professional skill acquisition. This is consistent with prior work showing that intrinsic motivation is associated with deeper engagement, persistence, strategic effort, and active participation in learning contexts (12–14).

The indirect association through intrinsic motivation was numerically larger than the indirect association through AI self-efficacy. Although this difference should be interpreted cautiously, it may suggest that students’ motivated learning depends strongly on whether future-oriented goals are internalized as personally meaningful and interesting. In other words, competence beliefs in AI-accessible environments may be important, but they may not be sufficient by themselves to sustain motivated learning unless students also experience learning as self-endorsed. This finding therefore reinforces self-determination theory by suggesting that autonomous motivation remains central to professional skill-oriented motivated learning, even in university contexts where AI-related tools are increasingly accessible.

Finally, the results supported the theoretically specified serial indirect pathway from future orientation to motivated learning through AI self-efficacy and intrinsic motivation. Although this pathway accounted for a relatively small proportion of the total effect, 3.83%, it remains theoretically meaningful because it links future-directed goal orientation, AI-related competence beliefs, and intrinsic motivation within one integrated motivational process. Future-oriented students may be more likely to view AI-related competence as relevant to their future academic and professional development. This interpretation may strengthen their AI self-efficacy by making AI-related learning demands appear more useful, manageable, and connected to long-term goals. In turn, AI self-efficacy may support intrinsic motivation by reducing uncertainty and enhancing students’ sense of competence in AI-accessible learning environments. From the perspective of self-determination theory, such competence-related beliefs can help students approach learning with greater curiosity, initiative, and self-endorsed engagement rather than simply responding to external demands (12, 14).

This serial pathway suggests that AI self-efficacy may be motivationally meaningful when it is connected to intrinsic motivation. In other words, students’ perceived capability in relation to AI technologies/products may not only be directly associated with motivated learning, but may also help create conditions under which students experience learning as more interesting, manageable, and personally meaningful. Intrinsic motivation may then translate this sense of competence into greater willingness, effort, and determination in professional skill acquisition. Importantly, the relatively small proportion of this serial indirect effect suggests that it should not be interpreted as the dominant explanation for motivated learning. Rather, it represents a supplementary but theoretically informative pathway showing how future orientation, AI-related competence beliefs, and intrinsic motivation may work together. Practically, this finding suggests that supporting students’ AI self-efficacy alone may be insufficient. Educators may also need to help students connect AI-related competence with their personal interests, future goals, and meaningful professional development. Given the cross-sectional design, this sequence should be interpreted as a theoretically specified pattern of associations rather than evidence of a confirmed temporal or causal process. Future longitudinal or experimental research is needed to examine the temporal ordering of these constructs more rigorously.

### 4.1 Theoretical and practical implications

Grounded in the motivation-cognition-behavior perspective and self-determination theory, this study offers three theoretical implications (3, 12). First, it extends research on future orientation by examining its association with motivated learning toward professional skill acquisition in a university context where AI-related technologies/products are increasingly accessible. Prior research has linked future orientation to academic engagement, self-regulation, persistence, and academic adjustment (4–7). The present findings extend this line of work by suggesting that future orientation may also be associated with students’ willingness and effort to acquire professional skills.

Second, the study clarifies the psychological relevance of AI self-efficacy within a broader motivational process. Rather than treating AI self-efficacy as a direct indicator of actual AI use or formal AI integration in coursework, this study conceptualized it as a domain-specific competence belief concerning students’ perceived capability in relation to AI technologies/products (10, 11). The results showed that AI self-efficacy statistically accounted for part of the indirect association between future orientation and motivated learning. This finding suggests that AI-related efficacy beliefs may be relevant to understanding students’ professional skill-oriented motivated learning, while remaining distinct from actual AI exposure or use.

Third, the study provides preliminary support for a theoretically specified pathway linking future orientation, AI self-efficacy, intrinsic motivation, and motivated learning. Future orientation was positioned as a distal motivational disposition, AI self-efficacy as a cognition-related competence belief, intrinsic motivation as a proximal motivational process, and motivated learning as a learning-related tendency involving effort, determination, and perceived importance of professional skill acquisition. This interpretation is consistent with self-determination theory, which emphasizes the role of competence-related beliefs and autonomous motivation in learning engagement (12–14). However, because the data were cross-sectional, this pathway should be interpreted as a theoretically grounded pattern of associations rather than as evidence of a confirmed temporal or causal process. Future studies should further examine these relationships using longitudinal or experimental designs.

From a practical perspective, the findings suggest several possible directions for supporting students’ motivated learning toward professional skill acquisition. First, educators may help students strengthen future orientation through structured goal-setting activities, reflective planning exercises, and career-aligned guidance that connect current learning with future academic or professional goals (2, 3). Second, students’ AI self-efficacy may be supported through guided and low-stakes opportunities to engage with AI-related technologies/products, accompanied by clear instructions, feedback, and peer or mentor support (10, 11). Third, intrinsic motivation may be encouraged through autonomy-supportive learning designs, such as meaningful choices, relevance to students’ interests, and opportunities to connect professional skill learning with personally meaningful goals (12–14). These practices should be understood as tentative implications based on cross-sectional associations rather than as evidence of direct intervention effects. In particular, the small but significant serial pathway suggests that AI self-efficacy may have greater motivational value when students can connect AI-related competence with intrinsic interest and personally meaningful future goals.

### 4.2 Limitations and future research

This study has several limitations that suggest directions for future research. First, the sample was drawn from a single university in Hubei Province, China, which may limit the generalizability of the findings. Future studies should include more diverse student populations across different regions, institutions, and cultural contexts to validate and extend the present results.

Second, the cross-sectional design restricts the ability to establish temporal ordering or causal relationships among the variables. Although the proposed serial pathway was theoretically grounded, the cross-sectional design cannot establish whether AI self-efficacy precedes intrinsic motivation or whether these constructs influence each other over time. Therefore, the proposed pathway should be interpreted as theoretically specified rather than temporally confirmed. Future studies should use longitudinal or experimental designs to examine the temporal ordering among future orientation, AI self-efficacy, intrinsic motivation, and motivated learning more rigorously.

Third, all variables were measured using self-report questionnaires, which may introduce common method variance and response bias. Although procedural remedies and Harman’s single-factor test were used as preliminary checks, common method variance cannot be fully ruled out (19, 22). In addition, although parcel-level CFA helped reduce model complexity, parcelling may obscure item-level misspecification, such as cross-loadings or correlated residuals. Future research could combine self-report data with behavioral indicators, learning analytics, teacher evaluations, or longitudinal records of students’ engagement with AI-related technologies/products, and further replicate the measurement model using item-level CFA.

Fourth, although this study focused on future orientation, AI self-efficacy, intrinsic motivation, and motivated learning, other psychological factors, such as emotion regulation, AI anxiety, technology acceptance, or perceived usefulness of AI tools, were not examined. Incorporating these variables into future models may provide a more comprehensive understanding of students’ motivated learning in university contexts where AI-related technologies/products are increasingly accessible (1, 21, 23).

## 5 Conclusion

This study provides preliminary evidence that future orientation is positively associated with motivated learning among university students, and that AI self-efficacy and intrinsic motivation statistically account for indirect associations between these two constructs. This finding is consistent with prior work suggesting that future-oriented students are more likely to connect present learning with long-term goals and regulate effort accordingly (2–4). The results further suggest that AI-related competence beliefs may be psychologically relevant within this broader motivational process, particularly in university contexts where AI-related tools are increasingly accessible (11, 12). However, because this study measured perceived AI self-efficacy rather than actual AI use or the effects of AI-based instruction, these AI-related implications should be interpreted cautiously. In the present sample, the indirect association through intrinsic motivation was numerically larger than that through AI self-efficacy, although this difference should be interpreted cautiously given the cross-sectional design. Overall, the findings offer tentative implications for educators who aim to support students’ future-oriented goal reflection, perceived competence in relation to AI technologies/products, and autonomous motivation.

## Data Availability

The datasets presented in this study can be found in online repositories. The names of the repository/repositories and accession number(s) can be found below: The data are available in the Harvard Dataverse repository: https://doi.org/10.7910/DVN/JWPUD0.
